# A Pilot Study to Evaluate the Acceptability of Using a Smart Pillbox to Enhance Medication Adherence Among Primary Care Patients

**DOI:** 10.3390/ijerph16203964

**Published:** 2019-10-17

**Authors:** Edmond Pui Hang Choi

**Affiliations:** School of Nursing, LKS Faculty of Medicine, 4/F, William M.W. Mong Block, 21 Sassoon Road, Pokfulam, Hong Kong; h0714919@connect.hku.hk

**Keywords:** patient acceptance of health care, medication adherence, primary health care

## Abstract

Smart pillboxes that remind patients to take medication may help avoid unintended non-adherence to medication regimens. To better understand the implementation potential of smart pillboxes among patients with chronic diseases, this study aimed to explore patients’ acceptability to use such devices and its associated factors. Five-hundred primary care patients aged 40 years or older were randomly recruited from a government-funded primary care clinic in Hong Kong. Patients were asked (i) if they needed to take medication daily, (ii) how many daily oral medications they needed to take on average, (iii) if they had ever missed a dose by accident, and (iv) if they were willing to use a smart pillbox for free to remind them to take medication. Out of the 344 participants included in the analysis who needed to take daily oral medication, 49.1% reported having previously missed a dose by accident, and 70.6% were willing to use a smart pillbox for free. A multiple logistic regression model found that male patients (adjusted odds ratio (aOR): 0.59) and patients with hypertension (aOR: 0.56) were less likely to have previously missed a dose by accident. Patients who needed to take a greater number of daily medications (aOR: 1.16), who had previously missed a dose by accident (aOR: 2.44), with heart disease (aOR: 3.67) and with a high monthly income (aOR: 2.30) were more willing to use a smart pillbox, while older patients (aOR: 0.95) were less willing to do so. Primary care patients who reported missing a dose by accident were 2.4 times as likely to want to use a smart pillbox while those with heart disease were almost 4 times as likely to want to use a smart pillbox. Further studies such as those evaluating the willingness to pay for smart pillboxes and randomised control trials to evaluate the effectiveness of smart pillboxes in enhancing medication adherence should be conducted to provide more evidence about the implementation potential of such devices.

## 1. Introduction

It is estimated that approximately one in three Chinese adults in Hong Kong have chronic diseases such as hypertension, diabetes and heart disease, with 59% of those being less than 60 years old [1]. The treatment of such diseases commonly includes the long-term use of medication. Adherence to medication regimens according to the recommendations of patients’ clinicians is of paramount importance in ensuring optimal patient outcomes. Non-adherence can lead to adverse outcomes such as failure to control blood pressure among patients with hypertension [2], increased risk of mortality in diabetes patients [3] and greater risk of cardiovascular mortality among patients with coronary artery disease [4]. Non-adherence can be intentional (i.e., an active decision on the part of patients to eschew prescribed treatments) or unintentional (i.e., a passive process whereby patients fail to adhere to the regimen through forgetfulness or carelessness) [5]. Unintentional non-adherence is common. A study in the United States found that in the preceding six months 62.4% of participants had forgotten to take medication, while 23.0% had been careless at times about taking medication [5]. There are a variety of strategies to improve mediation adherence. These include patient education and traditional devices such as weekly pill boxes [6]. Advances in technology have enabled the development of smart pillboxes that may help prevent unintended non-adherence [7]. Smart pillboxes can keep track of patients’ schedules and remind them to take the right medication, while some can even send alert notifications to caregivers. The use of smart pillboxes is an alternative to improve medication compliance among non-adherent patients [8,9].

A few studies have been conducted to specifically explore patients’ acceptability of new technology to enhance medication adherence. A study among HIV-positive patients in the United States found that nearly 70% of the respondents would like to use a technology-based intervention to help with HIV medication adherence [10]. A mixed method study on patients with coronary heart disease also supported the acceptability of the technology-based intervention to improve mediation adherence. The study found that most respondents appreciated that the smartphone apps could remind them to take their medication at the correct time, and they found the function very useful [11]. A study on older people in the United States found that 84% of the study participants expressed a desire to use technology-enhanced medication management device [12]. Previous studies suggest that patients have a high acceptability of using new technology to enhance medication compliance in general. 

The main purpose of this pilot study was to explore patients’ willingness to use smart pillboxes and its associated factors in order to better understand the implementation potential of such devices among patients with chronic diseases in primary care.

## 2. Materials and Methods

Five hundred primary care patients aged 40 years or older were randomly recruited from a government-funded primary care clinic in Hong Kong. After obtaining written informed consent, patients were asked (i) if they needed to take medication daily, (ii) how many daily oral medications they needed to take on average, (iii) if they had ever missed a dose by accident, and (iv) if they were willing to use a smart pillbox for free to remind them to take medication. The operational definition of the smart pill box in the present study was that daily medications were organized in the pill box (similar to the 7-day pill organizer) and one important feature was that the smart pill box could remind patients to take medication daily through their smartphone. A trained research assistant obtained written informed consent, administered the questionnaire and clarify questions raised by the study participants.

In addition to descriptive statistics, multiple logistic regression was used to explore factors associated with having previously missed a dose by accident and willingness to use a smart pillbox. Socio-demographic factors, three highly prevalent chronic diseases (hypertension, diabetes mellitus and heart disease) and the number of daily oral medications that patients needed to take were included in the regression model to explore factors associated with having missed a dose. To explore factors associated with willingness to use a smart pillbox, we added another variable asking if patients had ever missed a dose by accident in the regression. We chose hypertension, diabetes mellitus and heart disease as the only disease factors because a previous study involving the Hong Kong general population found that these three diseases are highly prevalent and more than 70% of patients with these diseases need to take regular medication [1]. In the analysis, age and the number of daily oral medications taken were continuous variables while others were categorical variables.

For sample size justification, we conservatively assumed that the prevalence of primary care patients who need to take daily oral medication was 50%, meaning that at least 385 primary care patients were needed to ensure 5% precision with a 95% confidence interval.

The study protocol was approved by the institutional review board: HKWC (UW19-179). Written informed consent was obtained from each participant.

## 3. Results

Out of the 500 randomly recruited patients, 344 needed to take daily oral medication and were thus included in the further analysis. The mean age of those patients was 59.9 years (standard deviation (SD): 9.7), and 55.2% were female. The prevalence of hypertension, diabetes mellitus and heart disease was 70.1%, 21.5% and 15.1%, respectively. The mean number of daily oral medications taken was 2.2 (SD: 2.6). Almost half (49.1%) the participants reported that they had previously missed a dose by accident, and 70.6% were willing to use a smart pillbox for free. Table 1 shows the results of descriptive statistics. Multiple logistic regression found that male patients (adjusted odds ratio (aOR): 0.59) and patients with hypertension (aOR: 0.56) were less likely to have previously missed a dose by accident. Patients who needed to take a higher number of daily medications (aOR: 1.16), who had previously missed a dose by accident (aOR: 2.44), with heart disease (aOR: 3.67) and with a high monthly income (aOR: 2.30) were more willing to use a smart pillbox, while older patients (aOR: 0.95) were less willing to do so. Table 2 shows the results of regression analysis to explore factors associated with having previously missed a dose by accident and willingness to use a smart pillbox. 

## 4. Discussion 

This study is the first to explore willingness to use smart pillboxes to enhance medication regimen adherence among primary care patients who take medication regularly. The high prevalence of unintended non-adherence implies a high unmet need to improve medication adherence, which can in turn lead to better treatment outcomes. About 70% of the study participants were willing to use smart pillboxes to remind them to take medication. The finding was comparable to those reported in the previous studies. Previous studies on older patients [12], HIV-positive patients [10] and patients with coronary heart disease [11] found a high acceptability of using new technology to remind them to take medication. The high willingness to use smart pillboxes suggests we can consider providing such devices to reduce unintended non-adherence among primary care patients. However, we still need to explore patients’ willingness to pay for such devices before the implementation. Besides, around 30% of the patients did not want to use such devices. Their reasons were not explored in the present study. Further qualitative studies can be conducted to explore why some patients would like to use smart pillboxes while some would not. 

Unsurprisingly, patients who needed to take a large number of daily medications and who had previously missed a dose were more willing to use such devices. Patients who had missed a dose might want a proactive strategy to avoid the recurrence of unintended non-adherence. Patients with heart disease were more willing to use such devices than patients with other chronic diseases. Adherence to increasingly complex treatment regimens represents a great effort for chronic heart disease patients [13]. Therefore, these patients may wish to use such devices to help them manage their medications and lower the treatment burden.

Some limitations should be noted. First, we only used a single item to assess medication adherence. The estimated prevalence might be biased. Further studies should use a validated assessment tool to evaluate medication adherence. Second, we only asked if our participants were willing to use the smart pillboxes for free. In fact, patients are likely to routinely “say yes” to free devices. Further studies should be conducted to evaluate “willingness to pay”. Moreover, qualitative studies should be considered to explore factors to enhance compliance in using such devices. Third, in the present study, we did not ask if they had previously used a smart pillbox because their prior experience might affect their willingness to use such devices subsequently. Fourth, the recruitment of primary care participants only may limit the generalisability of the findings to patients with more serious diseases such as cancer and HIV, who might be more cautious about medication compliance. Further studies could be conducted to explore if types of diseases will affect the willingness to use such devices. Nevertheless, most patients with chronic diseases who need to take long-term medication are managed in primary care doctors in many countries.

## 5. Conclusion

To conclude, primary care patients who reported missing a dose by accident were 2.4 times as likely to want to use a smart pillbox, while those with heart disease were almost 4 times as likely to want to use a smart pillbox. Further studies, such as those evaluating the willingness to pay for smart pillboxes, randomised control trials to evaluate the effectiveness of smart pillboxes in enhancing medication adherence and cost-effectiveness analysis should be performed to provide more evidence about the implementation potential of such devices in clinical settings.

## Figures and Tables

**Table 1:** Characteristics of participants and study outcomes (*n* = 344).

Socio-Demographics	
Mean Age (SD)	59.9 (9.7)
	*n* (%)
Gender	
Women	190 (55.2%)
Men	154 (44.8%)
Marital status	
Not married	90 (26.2%)
Married	254 (73.8%)
Employment status	
Not working	164 (47.7%)
Working	180 (52.3%)
Monthly income	
<HKD $20,000	194 (56.4%)
≥HKD $20,000	150 (43.6%)
Smoking status	
Non-smoker	322 (93.6%)
Current smoker	22 (6.4%)
Drinking status	
Non-drinker	239 (69.5%)
Current drinker	105 (30.5%)
Hypertension	241 (70.1%)
Diabetes	74 (21.5%)
Heart disease	52 (15.1%)
Mean number of daily oral medications taken (SD)	2.2 (2.6)
	*n* (%)
Previously miss a dose by accident	
Yes	169 (49.1%)
No	175 (50.9%)
Willingness to use a smart pillbox	
Yes	243 (70.6%)
No	101 (29.4%)

Abbreviation: SD: standard deviation; HKD: Hong Kong dollar.

**Table 2:** Results of multiple logistic regression.

**Model 1: Having Previously Missed a Dose by Accident**			
	aOR	95% CI	*p*-Value
Age^	1.01	(0.98, 1.04)	0.496
Male (vs. female)	0.59	(0.36, 0.97)	0.039
Married (vs. not married)	0.86	(0.51, 1.45)	0.576
Monthly income, ≥HKD$20,000 (vs. <HKD$20,000)	1.23	(0.75, 2.03)	0.410
Working (vs. not working)	0.86	(0.50, 1.48)	0.585
Current smoker (vs. non-smoker)	1.94	(0.74, 5.08)	0.179
Current drinker (vs. non-drinker)	1.32	(0.80, 2.17)	0.271
Having hypertension	0.56	(0.35, 0.91)	0.020
Having diabetes	1.41	(0.80, 2.48)	0.233
Having a heart disease	1.03	(0.53, 2.03)	0.925
The number of daily oral medications taken^	1.01	(0.92, 1.12)	0.802
Nagelkerke R^2^: 0.057			
**Model 2: Willingness to use a smart pillbox**			
	aOR	95% CI	*p*-value
Age^	0.95	(0.92, 0.99)	0.005
Male (vs. female)	0.88	(0.50, 1.57)	0.675
Married (vs. not married)	1.26	(0.69, 2.30)	0.457
Monthly income, ≥HKD$20,000 (vs. <HKD$20,000)	2.30	(1.28, 4.15)	0.005
Working (vs. not working)	0.72	(0.38, 1.36)	0.309
Current smoker (vs. non-smoker)	0.53	(0.17, 1.61)	0.262
Current drinker (vs. non-drinker)	0.80	(0.45, 1.42)	0.446
Having hypertension	1.44	(0.82, 2.56)	0.207
Having diabetes	1.42	(0.71, 2.85)	0.317
Having a heart disease	3.67	(1.29, 10.45)	0.015
The number of daily oral medications taken^	1.16	(1.01, 1.34)	0.040
Previously missed a dose by accident, yes (vs. no)	2.44	(1.43, 4.16)	0.001
Nagelkerke R^2^: 0.210			

Abbreviation: aOR: adjusted odds ratio; CI: confidence interval; HKD: Hong Kong dollar; ^ Age and the number of daily oral medications taken were continuous variables.
